# Personalized Web-Based Weight Loss Behavior Change Program With and Without Dietitian Online Coaching for Adults With Overweight and Obesity: Randomized Controlled Trial

**DOI:** 10.2196/17494

**Published:** 2020-11-05

**Authors:** Alline Beleigoli, Andre Q Andrade, Maria De Fatima Diniz, Antonio Luiz Ribeiro

**Affiliations:** 1 Flinders Digital Health Research Centre Flinders University Adelaide Australia; 2 Caring Futures Institute Flinders University Adelaide Australia; 3 Post Graduation Course of Adult Health Sciences Universidade Federal de Minas Gerais Belo Horizonte Brazil; 4 Quality Use of Medicines and Pharmacy Research Centre University of South Australia Adelaide Australia; 5 Department of Internal Medicine Universidade Federal de Minas Gerais Belo Horizonte Brazil; 6 Centre of Telehealth of the Hospital das Clinicas Universidade Federal de Minas Gerais Belo Horizonte Brazil

**Keywords:** obesity, overweight, healthy eating, diet, digital health, web platform, online coaching, personalized web interventions

## Abstract

**Background:**

The effect of computer- or human-delivered personalized feedback on the effectivess of web-based behavior change platforms for weight loss is unclear.

**Objective:**

We aimed to compare the effectiveness of a web-based behavior change intervention personalized through either computerized or human-delivered feedback with a nonpersonalized intervention in promoting weight loss in community-based adults with overweight or obesity.

**Methods:**

This pragmatic, 3-group, parallel-arm, randomized trial recruited students and staff in a Brazilian public university who were aged 18 to 60 years, had a BMI of ≥25 kg/m^2^, and were not pregnant. Participants were allocated to one of 3 groups: platform only (24-week behavior change program delivered using a web platform with personalized computer-delivered feedback), platform plus coaching (same 24-week web-based behavior change program plus 12 weeks of personalized feedback delivered online by a dietitian), or waiting list (nonpersonalized dietary and physical activity recommendations delivered through an e-booklet and videos). Self-reported weight at 24 weeks was the primary outcome. Changes in dietary and physical activity habits within 24 weeks were secondary outcomes.

**Results:**

Among the 1298 participants, 375 (28.89%) were lost to follow-up. In the intention-to-treat analysis, the platform-only and platform plus coaching groups had greater mean weight loss than the waiting-list group at 24 weeks (–1.08 kg, 95% CI –1.41 to –0.75 vs –1.57 kg, 95% CI –1.92 to –1.22 vs –0.66 kg, 95% CI –0.98 to –0.34, respectively). The platform-only and platform plus coaching groups, compared with the waiting list group, had a greater increase in the consumption of vegetables (3%, 95% CI 1% to 6% vs 5%, 95% CI 2% to 8% vs –3%, 95% CI –5% to 0%) and fruits (9%, 95% CI 6% to 12% vs 6%, 95% CI 2% to 9% vs 2%, 95% CI 0% to 6%) and a larger reduction in ultraprocessed food intake (–18%, 95% CI –23% to –13% vs –25%, 95% CI –30% to –20% vs –12%, 95% CI –16% to –8%). Changes in physical activity did not differ across the groups. Engagement was higher in the platform plus coaching group than in the platform-only group (7.6 vs 5.2 completed sessions; *P*=.007). Longer usage of the platform was associated with clinically meaningful (≥5%) weight loss (odds ratio 1.02, 95% CI 1.01 to 1.04).

**Conclusions:**

The web-based behavior change programs with computer- and human-delivered personalized feedback led to greater, albeit small-magnitude, weight loss within 24 weeks. Improvement in multiple dietary habits, but not physical activity, were also greater in the personalized programs compared with the nonpersonalized one. The human-delivered personalized feedback by the online dietitian coach increased user engagement with the program and was associated with a significantly higher chance of clinically meaningful weight loss.

**Trial Registration:**

ClinicalTrials.gov NCT03435445; https://clinicaltrials.gov/ct2/show/NCT03435445

**International Registered Report Identifier (IRRID):**

RR2-10.2196/10.1186/s12889-018-5882-y

## Introduction

Obesity is associated with a range of health complications and might lead to increased mortality [1]. Interventions that target healthy diet and physical activity behaviors are the cornerstones of weight management. Despite their limited success, particularly for weight loss maintenance in the long term, these interventions remain pivotal due to their additional benefits, such as diabetes and premature mortality prevention [2].

The World Health Organization estimates that 39% of adults worldwide are overweight and 13% are obese [3]. This prevalence translates into 650 million adults with obesity worldwide, which means that reducing overweight and obesity are key public health challenges. Interventions across different levels—individual, interindividual (social support by family and close relationships), and environmental—are essential to tackle the obesity epidemic [4]. However, the delivery of individual interventions, such as weight counseling within primary care, faces several barriers. System capacity, lack of confidence and knowledge among health professionals, uncomfortable feelings among people living with excessive weight about discussing the issue, and the limited timely access to health professionals such as dietitians are some of the challenges the health system faces in tackling obesity at the individual level [5].

In this context, digital health, which is defined as the use of information and communication technologies for health improvement, and particularly web-based programs, have the potential to reach a large number of people and be widely accessible and cost-effective [6]. Affordability, anonymity, and opportunity are additional advantages of web-based weight loss programs in comparison with traditional face-to-face interventions [7]. Despite all these potential benefits, results have been heterogeneous in regard to weight loss results. In a recent systematic review, we found that behavior change interventions delivered exclusively through the web led to clinically small benefits in the short term and no significant long-term weight loss when compared with offline interventions in overweight and obese adults [8]. This seems to be related to low long-term adherence to web-delivered weight loss interventions, similar to the problems faced by face-to-face interventions.

Understanding the multiple dimensions of behavior may be the key to improving adherence to and impact of behavior change interventions. The Behavior Change Wheel model identifies individual capability, opportunity, and motivation as interconnected dimensions of behavior that should be addressed for change [9]. This model translates well into the concept of personalized applications, in which users’ interactions with the application changes the experience and pathway to behavior change. In digital applications, personalization is usually enabled by algorithms but also by human-based guidance [10]. Health professional guidance, called “coaching” here, has been shown effective for behavior change in chronic obstructive pulmonary disease care [11] and heart failure improvement [12].

We aimed to investigate the impact of a personalized digital health behavior change intervention delivered exclusively via the web with and without online dietitian coaching on weight loss and on dietary and physical activity habits of people with overweight and obesity in the community compared with a minimal nonpersonalized intervention via the web. We also aimed to understand user engagement with the program.

## Methods

### Trial Design

The Online Platform for Healthy Weight Loss (POEmaS, from the abbreviation in Portuguese) study has been registered on ClinicalTrials.gov (NCT03435445), and the protocol with details of the intervention has been published elsewhere [13]. In brief, we conducted a 3-arm (1:1:1), parallel, randomized controlled trial, which recruited university students and staff in the Universidade Federal de Minas Gerais in Brazil. We used a pragmatic approach, with enrollment and outcomes assessment being exclusively online.

### Participants and Recruitment

University students and staff were invited through banners, posters, and mass media emails from September 25, 2017, to October 24, 2017. Participants were instructed to access a website, where they were informed about the aims of the study, inclusion criteria (aged 18 to 60 years, BMI ≥25 kg/m^2^, intention to lose weight through a behavior change program, and web access), and exclusion criteria (pregnancy, participation in any other weight loss program, or presence of conditions that demand specific dietary or physical activity recommendations, such as diabetes, heart failure, coronary artery disease, kidney disease, hepatic disease, cancer, phenylketonuria, celiac disease, food allergies, or bariatric surgery history).

### Randomization and Allocation

Those who were eligible were allocated to one of 3 study groups using a stratified randomized block design by sex and category of body mass index (25 to <30 or ≥30 kg/m^2^) using blocks of variable length (either 3 or 6). Then, participants received an email with information about the activities available to the group they were allocated to. The random allocation sequence and the algorithm for randomization were developed by a team of information technology specialists that did not participate in the recruitment or assessment processes. Those who did not complete the questionnaires about dietary and physical activity habits during the onboarding process could not proceed to the use of the platform.

### Study Groups

A detailed description of the rationale for the development of the intervention can be found elsewhere [14]. The waiting-list (control) group received a nonpersonalized minimal intervention based on dietary and physical activity recommendations delivered through a downloadable e-booklet and four 5-minute videos with information about health consequences of obesity, healthy dietary recommendations, healthy physical activity recommendations, and daily life strategies for the adoption of healthy behaviors. These resources were available to participants of this group through the platform from the beginning of the trial and could be accessed at any time. Moreover, like the intervention groups, this group received emails reminding them to report their weight and habits through the platform at 12 and 24 weeks after the trial baseline. By the end of the trial, these participants gained access to the weight loss program delivered through the web platform. This platform was adapted from a commercial software that has been used for multiple workforce behavior change and wellness interventions in Brazil.

The platform-only group was given access to a weight loss program delivered through the web-based platform. The program was based on diet [15] and physical activity [16] guidelines and on the Behavior Change Wheel model [9]. It comprised a total of 24 weekly sessions (12 weeks of an intensive program and 12 weeks of a maintenance program). The behavior change techniques (BCTs) [17] that were applied to address the capability, opportunity, and motivation of the participants in this group compared with the other groups can be seen in Multimedia Appendix 1. These BCTs were delivered using a range of software functionalities, such as short educational readings and videos, graphical and interactional tools, qualitative and quantitative (food diary) dietary monitoring, physical activity self-monitoring tasks, interactive games that created opportunities to invite friends and adopt healthy habits in daily life, and an online social network embedded in the platform and moderated by physicians and dietitians. Personalized feedback generated by a computational algorithm that took into account the goals set by each participant and the data on habits reported by the participant in initial questionnaires and through the self-monitoring tools was provided to participants from the fourth week of the intervention. This personalized feedback comprised feedback on behaviors and suggestions of strategies to improve their success in accordance with their individual goals. Furthermore, the platform suggested different modes of interaction (texts, social interaction, challenges) according to patterns of use during the first 4 weeks.

The platform and coaching group followed the same 24-week weight loss program delivered by the platform plus a 12-week initial course of online personalized education and feedback by a dietitian. The interactions between the participant and the dietitian could be initiated by either side through a private forum embedded in the platform. There was no limit to the amount of contact between them.

Although all groups received similar information about the target behaviors, there were substantive differences between the interventions received by the waiting-list group and the platform groups regarding the mode of delivery of the information and promotion of behavior change (Multimedia Appendix 1). An example of how the platform delivered the behavior change techniques can be demonstrated by the target behavior of increasing the intake of vegetables. The control group received information on the health consequences of this habit and instructions on the how to adopt this behavior through videos and recipes in the e-booklet. The capability of the groups using the platform was addressed through similar recommendations with short texts and videos. In addition, to address opportunity, these groups also received vegetable-rich recipes through the platform around the time of their main meals and were given challenges to post photos of vegetable-rich meals on the social media network. To enhance their motivation for this specific behavior, they scored points on their health score each time they reported vegetable intake on the data input tools on the platform or when they posted a photo in reference to a related challenge. An algorithm enabled them to receive tailored messages of feedback on that specific behavior based on the data they had input on the platform over the previous 4 weeks. Suggestions of resources available on the platform that could help them achieve their goals were also part of the feedback. For the platform plus coaching group, this process was enhanced by personalized feedback from the dietitian through a private chat forum.

The feedback provided by the coach included a review about the participant’s goal setting for behaviors and outcomes. Moreover, the dietitian specifically promoted self-monitoring of behaviors and outcomes, as well as emotional social support. When the participant had completed a food diary, the dietitian provided individualized feedback and informative social support regarding dietary quality and quantity. Reflective motivation was addressed by the development of action plans and problem-solving strategies pertinent to the individual’s circumstances. Prescripted responses to common topics were also used by the dietitian for feedback.

### Outcome Measures

We adopted a pragmatic approach by considering a real-world telehealth context and used self-reported (rather than measured) weight and BMI changes as the primary outcomes of the study. Weight reporting was required during baseline, and participants were encouraged through messages on the platform to continuously report it for the duration of the study. A validation study was conducted with a random sample of 12.5% of the study population to investigate the agreement between anthropometric measures that were self-reported by the participants and those that were measured by a trained research team using standardized and validated methods. Differences between self-reported and measured weight and BMI were clinically small and statistically nonsignificant, which led to high agreement between the self-reported weight and BMI and the measured anthropometry [18].

As secondary outcomes, the number of daily vegetable and fruit portions and the weekly consumption of sweetened beverages and ultraprocessed foods that were reported over the platform after 24 weeks were assessed through the Brazilian food frequency questionnaire (*questionario de frequência alimentar*) [19]. Moderate and vigorous physical activity was assessed by the Brief Physical Activity Assessment Questionnaire [20].

Outcomes assessors were blinded to group allocation.

### Sample Size

Based on 90% power to detect a significant difference of a 4 kg weight loss between groups, assuming the SD of weight would be 6.0 and using a 2-sided significance level of .05 and a 40% attrition rate, a sample size of 90 participants was calculated for each group [21].

### Data Analysis

Intention-to-treat analysis was performed for the primary outcomes at 12 and 24 weeks and for the secondary outcomes at 24 weeks. Analysis of covariance was used to test for differences in weight and BMI loss between groups at each time point, adjusted for treatment group as the predictor variable of interest and weight at baseline as a covariate. Statistical significance of the primary efficacy analysis (at 12 and 24 weeks) was adjusted for multiple testing procedures (Bonferroni). Analyses of the secondary outcomes were set at 24 weeks and used a 2-sided .05 significance level. Sensitivity analysis according to BMI (25-29.9 kg/m^2^ and ≥30 kg/m^2^) was performed.

Although not planned in the research protocol, due to the high number of missing values for the primary outcome, we performed multiple imputation by fitting logistic and linear regression models with both the predictors and the outcome as well as with other variables regarded as important to explain the missing values [19]. This procedure generated 5 complete data sets, which were used to estimate the association between group allocation and primary and secondary outcomes.

We compared clinically meaningful weight loss, defined as a ≥5% loss, weight stability (–5% to 5% difference), and weight gain (≥5% gain) across groups using chi-square tests. To investigate the association between adherence and clinically meaningful weight loss, we performed binary logistic regression with weight loss ≥5% (no or yes) at 24 weeks as the response variable and number of accesses to the platform, group (platform or platform plus coach), initial weight, and gender as covariates.

Data preprocessing and statistical analysis were done using the Python packages Pandas [22] and SciPy [23]. Multiple imputation was performed using IBM SPSS (version 18; IBM Corp).

### Ethics

The study was approved by the Ethics Committee of the Universidade Federal de Minas Gerais (CAAE: 73545717.5.0000.5149). All participants signed an online informed consent form prior to recruitment.

## Results

### Participant Characteristics

**Figure 1:**
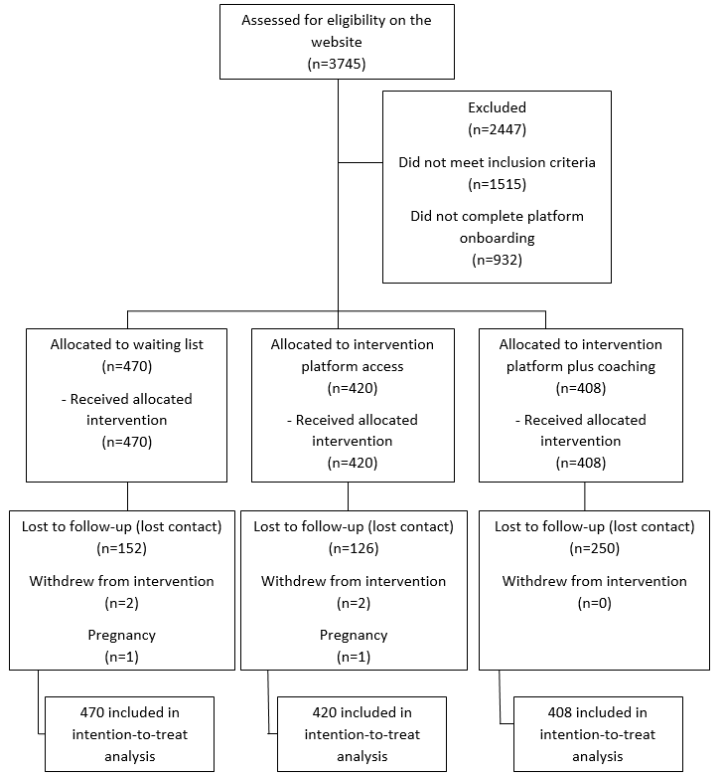
CONSORT flow diagram for the POEmaS randomized controlled trial. CONSORT: Consolidated Standards of Reporting Trials; POEmaS: Online Platform for Healthy Weight Loss.

A total of 3745 participants were assessed for eligibility, and 1298 were allocated to one of the 3 arms. This number is considerably higher than the sample size calculated for the study (n=270). We attribute this to a very successful recruitment process, which involved a mass communication strategy. Knowing from previous studies that interventions for weight loss, including web-based interventions, are usually associated with high dropout rates [8], we decided to increase our team capacity and resources to follow up with this higher number of participants. Across all groups, 375 of the 1298 participants (28.89%) were lost to follow-up. Participant workflow can be seen in Figure 1 and participants’ characteristics at baseline (N=1298) can be seen in Table 1.

**Table 1:** Participants’ characteristics at baseline.

Characteristic		Total (n=1298)	Waiting list (control) (n=470)	Platform only (n=420)	Platform and coaching (n=408)
Weight (kg), mean (95% CI)		82.8 (81.9-83.6)	82.6 (81.3-84.0)	83.4 (81.7-85.0)	82.3 (80.8-83.7)
Age (years), mean (95% CI)		33.6 (33.0-34.2)	33.4 (32.4-34.4)	34.4 (33.4-35.6)	33.0 (31.9-34.0)
BMI (kg/m^2^), mean (95% CI)		29.89 (29.66-30.13)	29.73 (29.37-30.08)	30.12 (29.67-30.58)	29.85 (29.44-30.26)
Female, n (%)		996 (76.7)	362 (77.0)	315 (75.0)	319 (78.2)
Vegetable intake^a^, mean (95% CI)		3.1 (3.1-3.2)	3.1 (3.0-3.2)	3.1 (3.0-3.2)	3.1 (3.0-3.2)
Fruit intake^a^, mean (95% CI)		2.8 (2.7-2.8)	2.8 (2.7-2.9)	2.7 (2.6-2.8)	2.8 (2.7-2.9)
Whole grains intake^a^, mean (95% CI)		1.8 (1.7-1.8)	1.7 (1.6-1.9)	1.7 (1.6-1.9)	1.8 (1.7-1.8)
Ultraprocessed foods^a^, mean (95% CI)		2.7 (2.7-2.8)	2.8 (2.7-2.9)	2.7 (2.6-2.8)	2.8 (2.6-2.8)
Sweetened beverages^a^, mean (95% CI)		1.8 (1.7-1.9)	1.8 (1.7-1.9)	1.8 (1.7-1.9)	1.8 (1.7-1.9)
Moderate physical activity^b^, mean (95% CI)		2.4 (2.2-2.6)	2.6 (2.4-2.8)	2.9 (2.5-3.0)	1.8 (1.6-2.0)
Vigorous physical activity^b^, mean (95% CI)		1.3 (1.1-1.8)	1.5 (1.1-1.8)	1.6 (1.3-2.0)	0.6 (0.4-1.3)
**Stages of change for physical activity^c^, n (%)**					
	Precontemplation	46 (3.9)	19 (4.0)	12 (2.9)	15 (3.7)
	Contemplation	435 (36.7)	146 (31.1)	163 (38.8)	126 (30.9)
	Preparation	276 (23.4)	86 (18.3)	96 (22.9)	94 (23.0)
	Action	256 (21.7)	86 (18.3)	79 (18.8)	91 (22.3)
	Maintenance	169 (14.3)	81 (17.2)	45 (10.7)	43 (10.5)

^a^Measured in servings per day.

^b^Measured as days per week exercising for more than 10 minutes.

^c^Precontemplation=not intending to engage in physical activity within 6 months; contemplation=intending to engage in physical activity within 6 months; preparation=intending to engage in physical activity within 30 days; action=physically active for less than 6 months; maintenance=physically active for more than 6 months.

### Primary Outcomes

**Figure 2:**
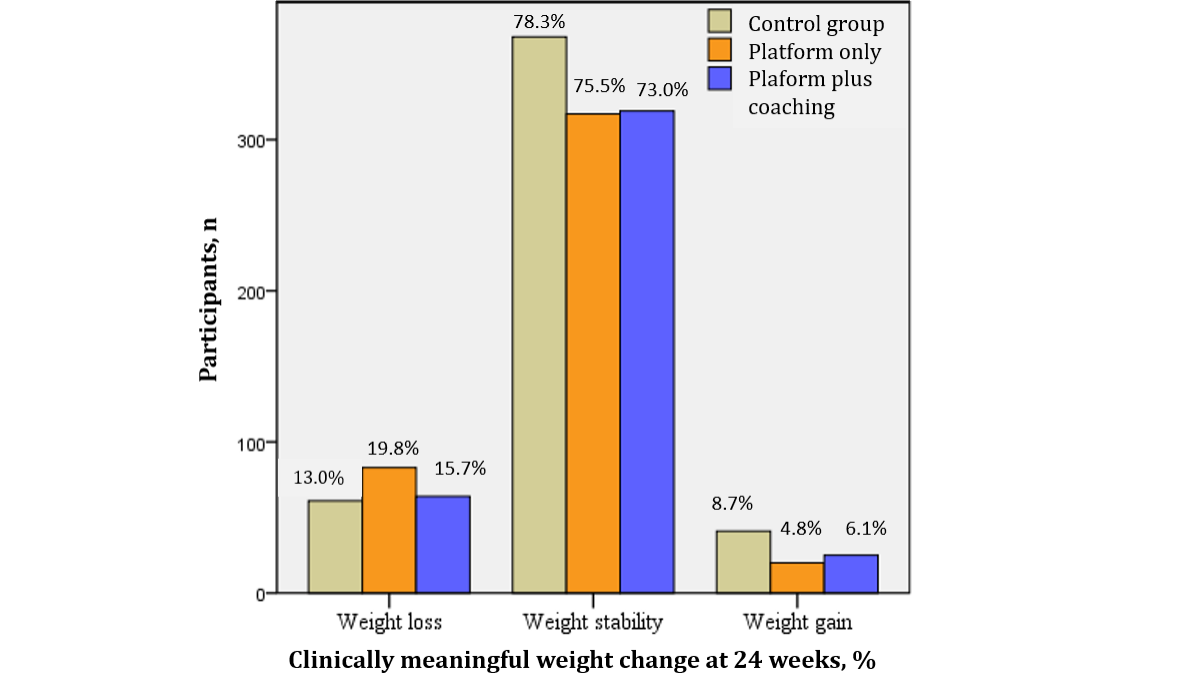
Clinically meaningful weight loss, stability, and gain per group at 24 weeks.

The absolute weight loss and BMI loss at 12 weeks were higher in the platform groups than in the waiting-list group, and there was no difference between the intervention groups (Table 2). At 24 weeks, weight loss and BMI loss were superior in the platform plus coaching group in comparison with the waiting-list group. A minimum 5% weight loss occurred more frequently in the platform-only (83/420, 19.8%) and platform plus coaching (64/408, 15.7%) groups than in the waiting-list group (61/270, 13.0%; *P*=.001), as seen in Figure 2. These results did not change when participants with overweight and obesity were analyzed separately (Multimedia Appendices 2 and 3) or when analysis included only participants with two or more weight reports (ie, no multiple imputation performed) (Multimedia Appendix 4).

**Table 2:** Primary outcomes after 12 and 24 weeks of follow-up according to intention-to-treat analysis.

Outcomes		Waiting list, mean (95% CI) (n=470)	Platform only, mean (95% CI) (n=420)	Platform plus coaching, mean (95% CI) (n=408)	*P* value^a^
**12 weeks**					
	Weight (kg)	82.06 (80.71 to 83.42)	82.23 (80.58 to 83.89)	80.88 (79.42 to 83.35)	N/A^b^
	Weight change (kg)	–0.56 (–0.83 to –0.30)	–1.14 (–1.42 to –0.85)	–1.36 (–1.65 to –0.80)	<.001
	BMI (kg/m^2^)	29.52 (29.15 to 29.89)	29.71 (29.25 to 30.18)	29.36 (28.93 to 29.78)	N/A
	BMI change (kg/m^2^)	–0.20 (–0.30 to –0.11)	–0.41 (–0.51 to –0.31)	–0.50 (–0.60 to –0.39)	<.001
**24 weeks**					
	Weight (kg)	81.97 (80.62 to 83.32)	82.29 (80.66 to 83.92)	80.68 (79.22 to 82.15)	N/A
	Weight change (kg)	–0.66 (–0.98 to –0.34)	–1.08 (–1.41 to –0.75)	–1.57 (–1.92 to –1.22)	.001
	BMI (kg/m^2^)	29.49 (29.12 to 29.86)	29.74 (29.29 to 30.20)	29.29 (28.86 to 29.72)	N/A
	BMI change (kg/m^2^)	–0.24 (–0.35 to –0.12)	–0.38 (–0.50 to –0.26)	–0.56 (–0.69 to –0.43)	.001

^a^*P* values based on comparisons across the 3 groups by analysis of covariance. For weight change at 12 weeks*,* *P* value for comparison between groups A (waiting list) and B (platform only) was .01, between groups A and C (platform plus coaching) was <.001, and between groups B and C was .80. For weight change at 24 weeks*,* *P* value for comparison between groups A and B was .23, between groups A and C was <.001, and between groups B and C was .14. For BMI change at 12 weeks, *P* value for comparison between groups A and B was .01, between groups A and C was <.001, and between groups B and C was .75. For BMI change at 24 weeks*,* *P* value for comparison between groups A and B was .28, between groups A and C was .001, and between groups B and C was .80.

^b^N/A: not applicable

### Secondary Outcomes

Both the platform-only and the platform plus coaching group had a greater increase in vegetable and fruit intake and a greater reduction in ultraprocessed food intake at 24 weeks in comparison with the control group. The reduction in sweetened beverage consumption was higher in the platform plus coaching group than in the platform-only group. Changes in other dietary habits and in moderate and physical activity duration were not different across groups (Table 3).

**Table 3:** Dietary and physical activity habits at 24 weeks and percent change from baseline (95% CI) across study groups.

Outcomes	Waiting list (control), mean (95% CI)	Platform only, mean (95% CI)	Platform plus coaching, mean (95% CI)	*P* value^a^
Vegetable intake (servings/day)	3.1 (3.0 to 3.2)	3.2 (3.1 to 3.3)	3.3 (3.2 to 3.4)	N/A^b^
Vegetable intake change^c^	–3 (–5 to 0)	3.0 (1 to 6)	5 (2 to 8)	.001
Fruit intake (servings/day)	2.9 (2.8 to 2.9)	3.0 (2.9 to 3.0)	2.9 (2.9 to 3.0)	N/A
Fruit intake change^c^	2 (–0 to 6)	9 (6 to 12)	6 (2 to 9)	.02
Whole grains (servings/day)	1.8 (1.7 to 1.9)	1.8 (1.7 to 1.9)	1.7 (1.6 to 1.8)	N/A
Whole grains change^c^	3 (–2 to 9)	2 (–1 to 10)	–5 (–12 to 2)	.11
Ultraprocessed foods (servings/day)	2.5 (2.4 to 2.6)	2.3 (2.2 to 2.4)	2.2 (2.1 to 2.3)	N/A
Ultraprocessed foods change^c^	–12 (–16 to –8)	–18 (–23 to –13)	–25 (–30 to –20)	.005
Sweetened beverages (servings/day)	1.7 (1.6 to 1.8)	1.8 (1.7 to 1.9)	1.6 (1.5 to 1.7)	N/A
Sweetened beverages change^c^	–6 (–12 to 0)	0 (–5 to 7)	–14 (–21 to –8)	.008
Moderate activity duration^d^	2.4 (2.2 to 2.6)	2.7 (2.4 to 2.9)	2.3 (2.1 to 2.5)	N/A
Moderate activity duration change^e^	–15 (–25 to –3)	–4 (–13 to –5)	23 (9 to 37)	.21
Vigorous activity duration^d^	1.3 (0.9 to 1.8)	1.6 (1.1 to 2.2)	1.1 (0.6 to 1.5)	N/A
Vigorous activity duration change^e^	–14 (–28 to 0)	2 (–9 to 13)	4 (–18 to 11)	.19

^a^*P* value based on analysis of covariance. For vegetable intake change, difference between groups A (waiting list) and B (platform only) was .03, between groups A and C (platform plus coaching) was .001, and between groups B and C was .71. For fruit intake change, difference between groups A and B was .01, between groups A and C was .49, and between groups B and C was .47. For ultraprocessed food intake change, difference between groups A and B was .35, between groups A and C was .003, and between groups B and C was .28. For sweetened beverage intake change, difference between groups A and B was .35, between groups A and C was .26, and between groups B and C was .01.

^b^N/A: not applicable.

^c^Percent change in servings per day from baseline.

^d^Measured as days per week exercising for more than 10 minutes.

^e^Percent change in number of days per week from baseline.

### Engagement

**Figure 3:**
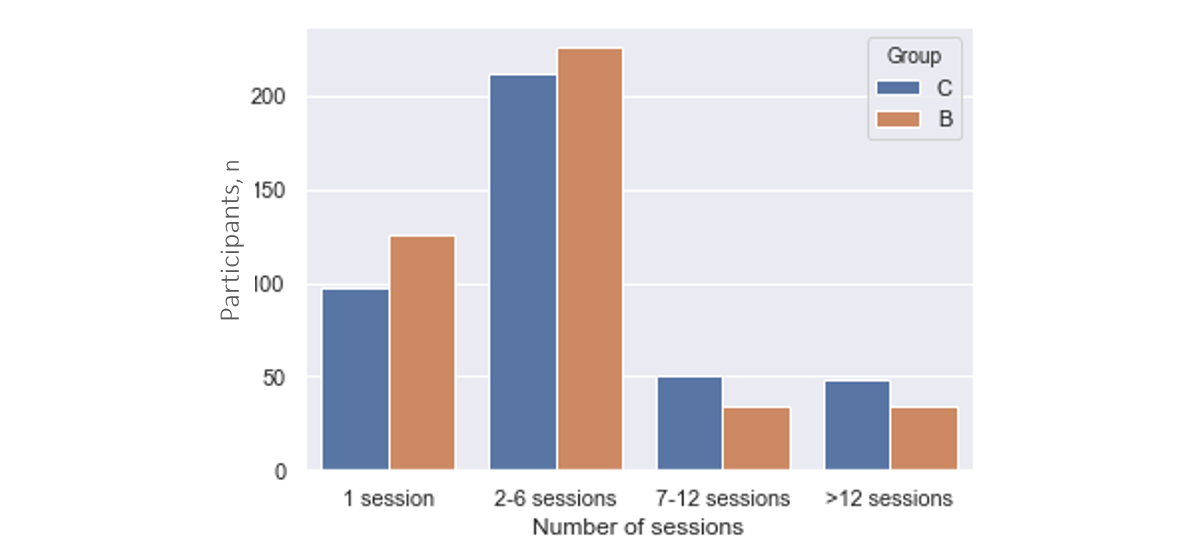
Number of participants completing sessions per group (group B is the platform-only group and group C is the platform plus coaching group) over 24 weeks.

The mean number of sessions completed was 5.2 (95% CI 4.1-6.3) and 7.6 (95% CI 6.0-9.1) for the platform-only group and the platform plus coaching group, respectively *(P=*.007). Participants’ interactions with the platform showed considerable initial attrition, which can be verified by the large number of participants who completed only 1 session: 126 of 420 (30.0%) in the platform-only and 97 of 408 (23.7%) in the platform plus coaching group. The number of participants who completed sessions at 12 and 24 weeks across all groups is shown in Figure 3. Except for whole grain intake, which was higher among completers, baseline characteristics of completers and noncompleters did not differ (Multimedia Appendix 5).

Functionalities that delivered self-monitoring of behavior and social support were the most accessed ones in both groups, with a higher mean access rate of the latter by the platform plus coaching group (48.7 times, 95% CI 37.8-59.6 vs 32.5 times, 95% CI 25.0-39.9; *P*=.02). The use of each platform functionality and the corresponding behavior change techniques delivered by the functionality are displayed in Table 4.

The total number of sessions completed by the participants was independently associated with clinically significant weight loss (≥5%) at 24 weeks (odds ratio 1.02, 95% CI 1.01-1.04) when adjusted for initial weight, study group (platform or platform plus coaching), and gender.

**Table 4:** Number of accesses to platform functionalities and the corresponding behavior change techniques between the platform-only and platform plus coaching groups.

Platform functionality (BCTs^a^)	Platform only, mean (95% CI)	Platform plus coaching, mean (95% CI)	*P* value
Weight report (self-monitoring of outcome of behavior)	2.7 (2.3-3.1)	3.3 (2.5-4.1)	.21
Behavior report (self-monitoring of behavior)	49.7 (27.1-72.3)	72.1 (43.5-100.6)	.23
Profile (goal setting of outcome; review outcome goals; feedback on behavior)	7.1 (5.6-8.5)	7.8 (6.2-9.5)	.49
Small texts (action planning; instruction on how to perform the behavior; information about antecedents, health consequences, and emotional consequences; reduce negative emotions; verbal persuasion about capability; restructuring the social environment)	5.8 (4.9-6.7)	5.9 (5.0-6.8)	.85
Challenges and gamification resources (problem solving; restructuring the social environment; avoidance and reducing exposure to cues for the behavior; imaginary reward)	0.2 (0.1-0.2)	0.2 (0.1-0.2)	.91
Online social network (unspecified, practical, and emotional social support; avoidance and reducing exposure to cues for the behavior)	32.5 (25.0-39.9)	48.7 (37.8-59.6)	.02

^a^BCT: behavior change technique.

## Discussion

### Primary and Secondary Outcomes

Weight and BMI loss were greater after 12 weeks and 24 weeks in the groups using the platform (with or without coaching) than in the group receiving a minimal intervention. The magnitude of weight loss (<2 kg) across the groups was small, which is similar to mean differences found in recent meta-analyses, in which weight loss programs delivered through the web were compared with face-to-face or to no interventions for people with overweight or obesity [8,24]. However, clinically meaningful weight loss (≥5%) was significantly more common in the platform groups with personalized feedback (83/420, 19.8% of the platform-only group and 64/408, 15.7% of the platform plus coaching group) than in the control group that received a nonpersonalized intervention (61/470, 13.0%). Moreover, compared with the control group, ultraprocessed food and sweetened beverage consumption in the platform groups decreased, while vegetable and fruit intake increased. This is a very positive result because only about 34.7% of Brazilian adults consume 5 or more servings per day of vegetables and fruits [25]. The importance of these results is also related to the fact that health benefits associated with vegetable and fruit consumption are independent of weight loss [26]. The superiority of the platform groups compared with the control group for both primary and secondary outcomes suggests that different behavior change techniques that address capability, opportunity, and motivation have a greater effect on promoting dietary behavior change and weight loss than a nonpersonalized focus on capability only [27].

Although our study design cannot point to which BCTs explain the difference in effectiveness across the groups, the BCTs of social support, personalized feedback, and self-monitoring of behaviors, which only the platform groups received, were important intervention differences. The efficacy of these BCTs in promoting weight loss has been reported by other studies [28].

Similar to other studies, there was no difference in short-term weight loss [29] and in changes in diet and physical activity between the group that received computer-based personalized feedback (platform only) and the group that also received the human-delivered personalized feedback (platform plus coaching). Despite this lack of differences in the outcomes, the addition of a health professional coaching service increased platform usage. This suggests that the feeling of having a human factor [10,30] or of being supervised [31] increases engagement, which might be particularly important for long-term weight maintenance [29].

### Engagement

User engagement results were similar to other large-scale weight loss interventions via the web [8]. Most of the losses to follow-up occurred at the beginning of the intervention across all groups, particularly in the waiting-list group (152/470, 32.3%). The study design mimicked real-world recruitment and usage conditions. In this sense, participants received email reminders to report their weight but not to use the platform, and there were no financial incentives or contact between the research team and the participants. The broad recruitment strategy led to the enrollment of a large proportion of individuals who were probably not predisposed to engage, as suggested by the high proportion of individuals (481/1298, 37.06% across all groups) who reported being on the precontemplation or contemplation stage of change for physical activity, according to the transtheoretical model–based questionnaire given to all participants at baseline (Table 1). A recruitment based on the stages of readiness to change for weight loss might have yielded different results [32]. Moreover, the mandatory completion of questionnaires in the dashboard before being able to use the platform and the technical issues in the beginning of the intervention (despite being promptly corrected) might have contributed to the loss of participants who were not highly motivated.

Our results also suggest that adherence to the behavior change intervention is key to the weight loss outcome. Our analysis showed that each additional session completed by participants was associated with a 2% increase (95% CI 1%-4%) in the chance of achieving clinically significant weight loss at 24 weeks. This demonstrates that engagement is a key factor for the success of online interventions, as reported by other studies, including those for long-term weight maintenance [10,33,34].

### Implications to Practice

The intervention delivery package, which involved broad recruitment and few follow-up visit requirements, mimics the conditions of a low-demand telehealth intervention in communities (rather than for patients in specific health care settings). In this context, a behavior change program delivered through a web platform might be an effective solution for public health interventions that promote weight loss and increased fruit and vegetable intake in the short term.

The inclusion of a human-based personalization strategy using an online dietitian coach did not appear to be more effective than the computer-based personalization strategy after 6 months of follow-up. This suggests that web platforms enhanced by algorithm-generated personalized feedback might be a good strategy for tackling overweight and obesity through lifestyle habit changes in the short term. This can be particularly useful in contexts in which the demand for professionals to support people with excessive weight cannot be met. However, since the human-delivered feedback led to higher rates of engagement and longer use of the platform, the human-delivered feedback strategy might be useful for participants with a high risk of abandoning the program, such as those reporting low levels of preintervention motivation or with multiple weight loss attempts [31].

### Strengths and Limitations

The large scale of this clinical trial (1298 participants) and its pragmatic nature are main strengths of this study. To our best knowledge, this is the largest trial evaluating health professional coaching for behavior change in a large and diverse group of participants recruited in the community. Such scale was only possible with an open recruitment strategy, which reduces the barrier for enrollment but also brings some limitations. The first limitation is related to not being able to take standardized and repeated measurements of weight and BMI, since we had no direct contact with most participants. Additionally, the large number of noncompleters, which is common to web-based interventions, increased the risk of bias in our results.

### Conclusion

A behavior change program for weight loss delivered through a web-based platform led to greater weight loss, increased fruit and vegetable intake, and reduced ultraprocessed food consumption compared with a minimal intervention, as measured up to 6 months into the intervention. The platform enhanced by human-delivered personalized feedback was not superior to the platform with a computer-based personalized approach for weight loss. However, it led to higher levels of engagement, which were associated, albeit weakly, with higher odds of achieving clinically significant weight loss.

## Appendix

Multimedia Appendix 1 Behavior change techniques (BCTs) across the study groups.

Multimedia Appendix 2 Primary outcomes analysis for participants with overweight.

Multimedia Appendix 3 Primary outcomes analysis for participants with obesity.

Multimedia Appendix 4 Primary outcomes at 12 and 24 weeks for participants with more than one report of weight.

Multimedia Appendix 5 Comparison of baseline characteristics of participants who completed and those who did not complete the intervention.

## Abbreviations

BCT: behavior change technique
POEmaS: Online Platform for Healthy Weight Loss
