# Supplementary material for: Personalized Web-Based Weight Loss Behavior Change Program With and Without Dietitian Online Coaching for Adults With Overweight and Obesity: Randomized Controlled Trial
Source: J Med Internet Res. 2020 Nov 5;22(11):e17494. doi: 10.2196/17494 (PMC7677024; doi:10.2196/17494)
Supplement: Multimedia Appendix 1 [file jmir_v22i11e17494_app1.docx]

Multimedia Appendix 1- Behavior change techniques (BCTs) across the study groups

| Control group | Intervention (Platform and Platform + coach) |
| --- | --- |
| 1.2. Problem solving  1.3. Goal setting (outcome)  1.4. Action planning  4.1. Instruction on how to perform the behavior  4.2 Information about antecedents  5.1. Information about health consequences  12.1 Restructuring the physical environment | 1.1 Goal setting (behavior)  1.2. Problem solving  1.3. Goal setting (outcome)  1.4. Action planning  1.5 Review behavior goal(s)  1.7. Review outcome goal(s)  2.2 Feedback on behavior  2.3. Self-monitoring of behavior  2.4. Self-monitoring of outcome(s) of behavior  3.1. Social support (unspecified)  3.2. Social support (practical)  3.3. Social support (emotional)  4.1. Instruction on how to perform the behavior  4.2 Information about antecedents  5.1. Information about health consequences  5.6. Information about emotional consequences  11.2 Reduce negative emotions  12.1 Restructuring the physical environment  12.2 Restructuring the social environment  12.3 Avoidance/reducing exposure to cues for the behavior  13.2 Framing/reframing  15.1 Verbal persuasion about capability  16.3 Imaginary reward |

*Codes according to the Behavior Change Techniques Taxonomy V1
