# Supplementary material for: Personalized Web-Based Weight Loss Behavior Change Program With and Without Dietitian Online Coaching for Adults With Overweight and Obesity: Randomized Controlled Trial
Source: J Med Internet Res. 2020 Nov 5;22(11):e17494. doi: 10.2196/17494 (PMC7677024; doi:10.2196/17494)
Supplement: Multimedia Appendix 2 [file jmir_v22i11e17494_app2.docx]

Multimedia Appendix 2- Primary outcomes analysis for participants with overweight

| Outcomes | Waiting list (control) (n=290) | Platform only  (n=256) | Platform/ coaching (n=247) | p value* |
| --- | --- | --- | --- | --- |
| Weight at baseline | 74.9 (73.9, 76.0) | 74.1 (73.1, 75.2) | 74.4 (73.3, 75.4) | N/A |
| BMI at baseline | 27.21 (27.04, 27.37) | 27.12 (  26.94, 27.30) | 27.15 (26.97, 27.34) | N/A |
| **12 weeks** |  |  |  |  |
| Weight (kg) | 74.3 (73.9, 74.6) | 73.4 (73.0, 73.7) | 73.2 (72.8, 73.6) | N/A |
| Weight change (kg) | -0.20 (-0.51, 0.12) | -1.10 (-1.45, -0.76) | -1.29 (-1.70,-0.89) | <0.001 |
| BMI (kg/m2) | 27.09 (26.96, 27.21) | 26.76 (26.63, 26.89) | 26.69 (26.82, 26.56) | N/A |
| BMI change (kg/m2) | -0.08 (-0.19, 0.04) | -0.40 (-0.53, -0.28) | -0.47 (-0.62,-0.33) | <0.001 |
| Weight loss (%) | 19 (6.6) | 41 (16.0) | 50 (20.2) | N/A |
| Weight stability (%) | 256 (88.3) | 202 (78.9) | 189 (76.5) | N/A |
| Weight gain (%) | 15 (5.2) | 13 (5.1) | 8 (3.2) | N/A |
| **24 weeks** |  |  |  |  |
| Weight (kg) | 74.1 (73.8, 74.5) | 73.2 (72.8, 73.7) | 73.3 (72.9, 73.7) | N/A |
| Weight change (kg) | -0.34 (-0.72, 0.03) | -1.24 (-1.64, -0.85) | -1.20 (-1.66, -0.75) | 0.002 |
| BMI (kg/m2) | 27.00 (26.82, 27.10) | 26.80 (26.69, 26.99) | 26.57 ( 26.43, 26.73) | N/A |
| BMI change (kg/m2) | -0.13 (-0.26, 0.01) | -0.45 (-0.60, -0.31) | -0.44 (-0.61, -0.27) | 0.001 |

a Mean (95% confidence interval)

b p values based on comparisons across the 3 groups by analysis of covariance.

For weight change at 12 weeks, p value for comparison between groups A (Waiting list) and B (Platform only)= 0.001, groups A and C (Platform/coaching) <0.001, groups B and C= 1.00; For weight change at 24 weeks, p value for comparison between groups A and B= 0.002, groups A and C 0.001, groups B and C=1.00; For BMI at 12 weeks, p value for comparison between groups A and B=0.001, groups A and C <0.001, groups B and C= 1.00; For BMI at 24 weeks, p value for comparison between groups A and B= 0.001, groups A and C=<0.001, groups B and C= 1.00
