# Supplementary material for: Personalized Web-Based Weight Loss Behavior Change Program With and Without Dietitian Online Coaching for Adults With Overweight and Obesity: Randomized Controlled Trial
Source: J Med Internet Res. 2020 Nov 5;22(11):e17494. doi: 10.2196/17494 (PMC7677024; doi:10.2196/17494)
Supplement: Multimedia Appendix 3 [file jmir_v22i11e17494_app3.docx]

Multimedia Appendix 3- Primary outcomes analysis for participants with obesity

| Outcomes | Control group (n=180) | Platform access  (n=164) | Platform plus coach (n=161) | p value* |
| --- | --- | --- | --- | --- |
| Weight at baseline | 95.1 (93.1, 97.1) | 97.8 (95.1, 100.5) | 94.4 (92.2, 96.6) | N/A |
| BMI at baseline | 33.79 (33.30, 34.27) | 34.81 (34.15, 35.47) | 33.99 (34.57, 33.41) | N/A |
| **12 weeks** |  |  |  |  |
| Weight (kg) | 95.3 (94.9, 95.7) | 94.5 (94.1, 94.9) | 94.2 (93.8, 94.7) | N/A |
| Weight change (kg) | -0.46 (-0.87, -0.06) | -1.21 (-1.63, -0.78) | -1.51 (-1.94,  -1.07) | 0.002 |
| BMI (kg/m2) | 34.01 (33.87, 34.16) | 33.74 (33.58, 33.89) | 33.64 (33.48, 33.79) | N/A |
| BMI change (kg/m2) | -0.20 (-0.32, -0.02) | -.0.45 (-0.60 -0.29) | -0.55 (-0.70, -0.39) | 0.001 |
| **24 weeks** |  |  |  |  |
| Weight (kg) | 95.1 (94.6, 95.7) | 94.52 (93.6, 94.8) | 94.5 (93.9, 95.0) | N/A |
| Weight change (kg) | -0.58 (-1.12, -0.04) | -1.58 (-2.13, -1.01) | -1.25 (-1.85, -0.65) | 0.05 |
| BMI (kg/m2) | 33.95 (33.76, 34.15) | 33.63 (33.43, 33.84) | 33.73 (33.53, 33.94) | N/A |
| BMI change (kg/m2) | -0.22 (-0.42, -0.02) | -0.57 (-0.77, -0.37) | -0.45 (-0.66, -0.24) | 0.047 |

a Mean (95% confidence interval)

b p values based on comparisons across the 3 groups by analysis of covariance.

For weight change at 12 weeks, p value for comparison between groups A (Waiting list) and B (Platform only)= 0.002, groups A and C (Platform/coaching)=0.002, groups B and C= 0.800; For weight change at 24 weeks, p value for comparison between groups A and B= 0.05, groups A and C=0.047, groups B and C=1.00; For BMI at 12 weeks, p value for comparison between groups A and B=0.002, groups A and C =0.002, groups B and C= 1.00; For BMI at 24 weeks, p value for comparison between groups A and B= 0.05, groups A and C=0.047, groups B and C= 0.804
