# Supplementary material for: Personalized Web-Based Weight Loss Behavior Change Program With and Without Dietitian Online Coaching for Adults With Overweight and Obesity: Randomized Controlled Trial
Source: J Med Internet Res. 2020 Nov 5;22(11):e17494. doi: 10.2196/17494 (PMC7677024; doi:10.2196/17494)
Supplement: Multimedia Appendix 4 [file jmir_v22i11e17494_app4.docx]

Multimedia Appendix 4- Primary outcomes at 12 and 24 weeks for participants with more than one report of weight

| Outcomes | Control group | Platform access | Platform plus coaching | p value* |
| --- | --- | --- | --- | --- |
| **12 weeks** |  |  |  |  |
| Number of participants included in this sub-analysis | 132 | 115 | 134 |  |
| Weight change† (kg) | -0.44(2.85) | -1.38 (2.76) | -1.05 (3.16) | 0.038 |
| BMI change† (kg/m2) | -0.16 (1.03) | -0.51 (1.03) | -0.37 (1.12) | 0.032 |
| **24 weeks** |  |  |  |  |
| Number of participants included in this subanalysis | 197 | 159 | 178 |  |
| Weight change† (kg) | -0.83 (3.99) | -1.15 (3.56) | -1.22 (3.88) | 0.587 |
| BMI change† (kg/m2) | -0.29 (1.40) | -0.43 (1.28) | -0.43 (1.35) | 0.553 |
