# Supplementary material for: Personalized Web-Based Weight Loss Behavior Change Program With and Without Dietitian Online Coaching for Adults With Overweight and Obesity: Randomized Controlled Trial
Source: J Med Internet Res. 2020 Nov 5;22(11):e17494. doi: 10.2196/17494 (PMC7677024; doi:10.2196/17494)
Supplement: Multimedia Appendix 5 [file jmir_v22i11e17494_app5.docx]

Multimedia Appendix 5- Comparison of baseline characteristics of participants who completed and those who did not complete the intervention

| Baseline characteristic | Completers | Non-completers | P value |
| --- | --- | --- | --- |
| Weight (kg) † | 82.4 (15.6) | 83.00 (15.6) | 0.458 |
| BMI (kg/m^2^) † | 29.7 (4.2) | 30.0 (4.4) | 0.311 |
| Age (years) † | 34.5 (11.1) | 33.0 (10.4) | 0.016 |
| Female gender (%) | 759 (78.8%) | 253 (75.4%) | 0.149 |
| Vegetables intake † (servings/day) | 3.2 (0.9) | 3.1 (1.0) | 0.130 |
| Fruit intake (servings/day) † | 2.9 (1.0) | 2.7 (1.0) | 0.061 |
| Whole grains intake † (servings/day) | 1.9 (1.3) | 1.6 (1.3) | <0.001 |
| Sweetened beverages † (servings/day) | 1.7 (1.2) | 1.9 (1.1) | 0.109 |
| Ultra-processed food † (servings/day) | 2.8 (1.0) | 2.8 (1.0) | 0.999 |
| Moderate physical activity † (days/week exercising more than 30 min) | 2.4 (2.2) | 2.3 (2.1) | 0.085 |
| Vigorous physical activity † (days/week exercising more than 20 min) | 1.4 (1.8) | 1.3 (1.5) | 0.527 |

† Mean (standard deviation)

P values based on t test for continuous variables and Chi-square for categorical variables.
